# Rapid progression of high-risk proliferative diabetic retinopathy induced by insulin intensive therapy: A case report

**DOI:** 10.1097/MD.0000000000024379

**Published:** 2021-02-19

**Authors:** Shuya Wang, Dezheng Liu, Xiaoyan Zhang, Hongsheng Bi

**Affiliations:** a Department of Ophthalmology, Shandong University of Traditional Chinese Medicine; b Department of Ophthalmology, Affiliated Eye Hospital of Shandong University of Traditional Chinese Medicine; c Department of Ophthalmology, Shandong Provincial Key Laboratory of Integrated Traditional Chinese and Western Medicine for Prevention and Therapy of Ocular Diseases; d Key Laboratory of Integrated Traditional Chinese and Western Medicine for Prevention and Therapy of Ocular Diseases in Universities of Shandong; e Eye Institute of Shandong University of Traditional Chinese Medicine, Jinan, China.

**Keywords:** case report, insulin intensive treatment, progressing of diabetic retinopathy, proliferative diabetic retinopathy

## Abstract

**Rationale:**

To summarize and analyze a case of rapid progression of high-risk proliferative diabetic retinopathy after the insulin intensive therapy (IT).

**Patient concerns:**

A 58-year-old type 2 diabetes female patient suffered a rapid and dramatic decline of vision acuity in the left eye in 2 months after the insulin IT. However, the best corrected visual acuity (BCVA) of her right eye, which was in much severer condition and received panretinal photocoagulation (PRP) before, improved after the IT.

**Interventions:**

The patient received intravitreal injection of conbercept (IVC) to her left eye, and 5 days later, underwent pars plana vitrectomy (PPV), combined with PRP and silicon oil injection.

**Outcomes:**

The postoperative BCVA of the left eye was 20/200 and improved to 20/160 one month later. During the subsequent 2 months of follow-up, her BCVA remained 20/160 in both eyes. Her blood glucose level also remained stable.

**Lessons:**

Insulin IT for untreated proliferative diabetic retinopathy (PDR) patients can cause severe and irreversible consequences, so for such patients, the conservative treatment for glycemic control may be much safer. But if insulin IT is inevitable, the patient should undergo PRP promptly before the IT, and close eye monitoring during the IT is also essential.

## 1 Introduction

Diabetic retinopathy (DR) is a leading cause of irreversible blindness in the world. It has been proven in some studies that intensive treatment (IT) to reduce glycemic levels, can slow down the progression of DR in both type 1^[1]^ and 2^[2]^ diabetes in the long-term development. Therefore, near-normalization of blood glucose level is a major target of DR monitoring. However, since first being noticed in 1983,^[3]^ clinical trials have demonstrated that insulin IT can cause a paradoxical early aggravation of DR called “ early worsening of diabetic retinopathy” (EWDR) in type 1^[2,4,5]^ and type 2 diabetes.^[6,7]^ Most patients at baseline in these studies were with minimal-to-moderate non-proliferative diabetic retinopathy (NPDR) or even without DR. And the cases of EWDR caused by insulin IT at a baseline of high-risk proliferative diabetic retinopathy (PDR) in type 2 diabetes patients were barely reported. Here, we report a case, in which a type 2 diabetes patient suffered an acute aggravation of pre-existing high-risk PDR after the insulin IT.

## 2 Case report

**Figure 1:**
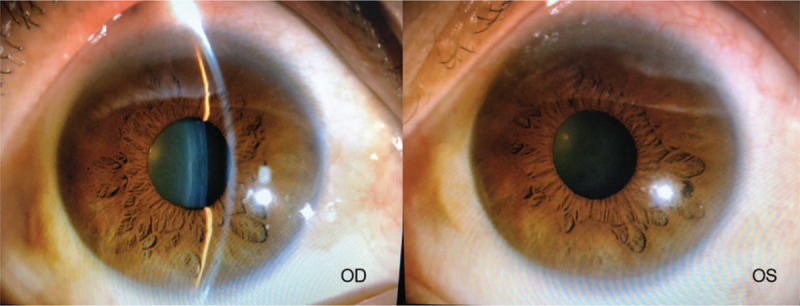
Slit lamp examination of anterior segment was normal except for the mild nuclear opacity of the bilateral lens.

**Figure 2:**
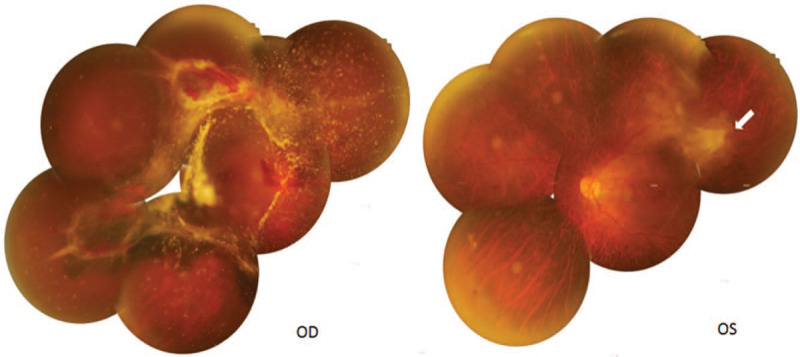
Fundus photography showed stellate vitreous particles and massive fibrovascular fibrous membranes extending to every quandrant of the retina in the right eye, and limited pre-retinal fibrous membrane adhering to the supratemporal quadrant of retina in the left eye.

**Figure 3:**
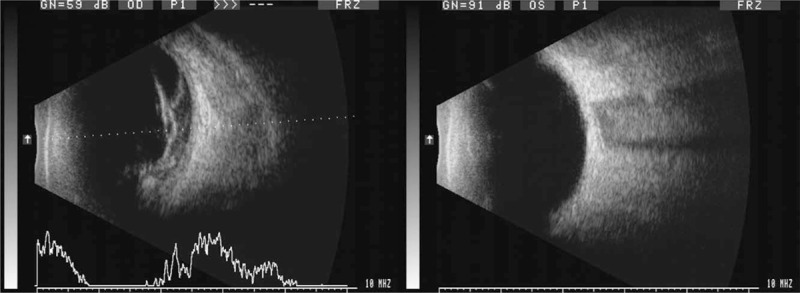
B-scan ultrasound images displayed the “table top” shaped tractional retinal detachment (TRD) in the right eye and partial pre-retinal fibrous membrane in the left.

**Figure 4:**
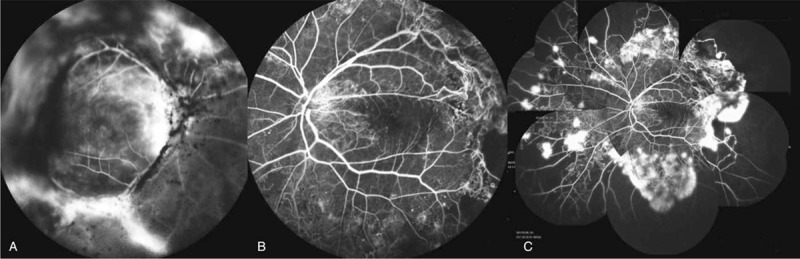
FA of the right (A) and left (B and C) eye. (A) FA of the right eye showed staining of the aggresive fibrovasluar membrane, traction on retinal blood vessels, and ischemic retinal vessels. (B and C) FA of the left eye revealed large areas of retinal nonperfusion, neovascularization of disc (NVD), and neovascularization elsewhere (NVE).

**Figure 5:**
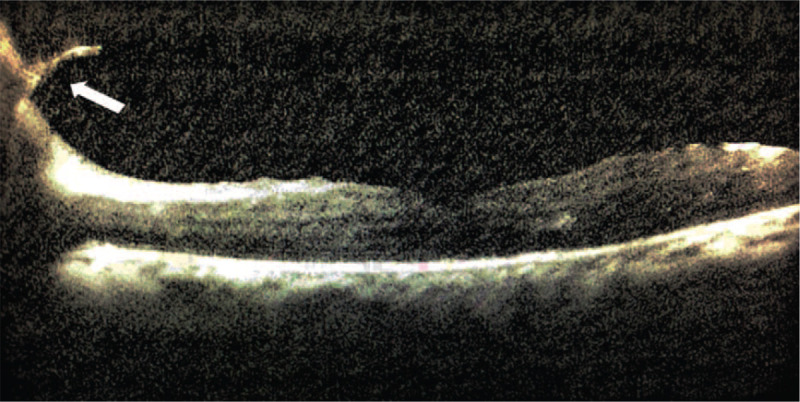
OCT of the left eye demonstrated the fibrous membrane on the optic disc (arrow) and relatively intact structure of the macula.

A 58-year-old Chinese woman was admitted to our hospital on 9th of January 2018 for the progressive visual loss of the right eye for 1 year. She reported a 21-year history of type 2 diabetes mellitus with poor glycemic control, and denied any other systemic diseases. She had been taking oral hypoglycemic drugs without insulin intervention since 20 years ago. Laboratory investigation revealed fasting blood-glucose 11.91 mmol/L, HbA1c 11%, and the serum cholesterol 2.9 mmol/L. The best-corrected visual acuity (BCVA) was hand motion in the right eye and 20/40 in the left. The intraocular pressures (IOP) were within normal limits in both eyes. Slit lamp examination of anterior segment was normal except for the mild nuclear opacity of the bilateral lens (Fig. 1). Fundus photography showed stellate vitreous particles, massive fibrovascular membrane extending to every quandrant of the retina in the right eye, and partial fibrous membrane adhering to the supratemporal quadrant of retina in the left eye (Fig. 2). B-scan ultrasound images displayed the “table top” shaped tractional retinal detachment (TRD) in the right eye and limited pre-retinal fibrous membrane in the left eye (Fig. 3). Fluorescein angiography (FA) showed staining of the aggressive fibrovascular membrane and ischemic retinal vessels in the right eye (Fig. 4A). And the FA of the left eye was not that optimistic as what has been shown in the fundus photography. It revealed large areas of retinal nonperfusion, neovascularization of disc (NVD), and neovascularization elsewhere (NVE) (Fig. 4B and C), which can be defined as the manifestation of high-risk PDR according to the Diabetic Retinopathy Study.^[8]^ Cross-sectional optical coherence tomography (OCT) of the left eye demonstrated the fibrous membrane on the optic disc and relatively intact structure of the macula, which explained the relative good visual acuity of the left eye (Fig. 5). The diagnosis of PDR in both eyes was established according to the examinations mentioned above. During hospitalization, a 3-port 25-G pars plana vitrectomy (PPV) was performed in the patient's right eye, combined with panretinal photocoagulation (PRP) and silicon oil injection. The postoperative BCVA of the right eye was 20/1000. Although the fundus laser therapy should be given to the patient's left eye as per the Early Treatment Diabetic Retinopathy Study,^[9]^ she refused for fear of the temporary blurred vision.

**Figure 6:**
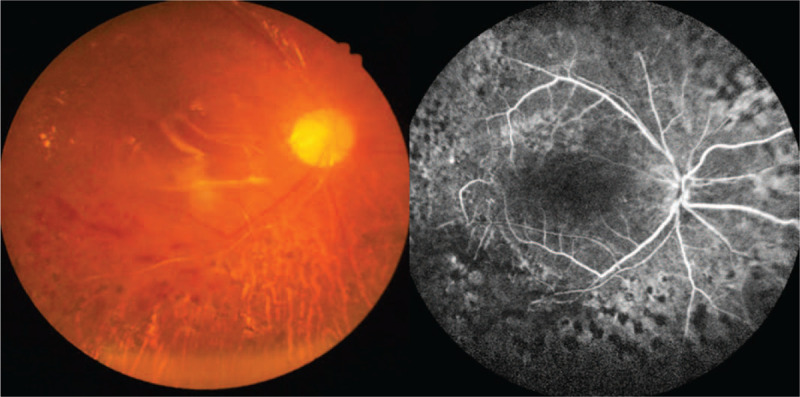
Fundus photography and FA of the right eye 2 months after the PPV demonstrated the stable condition of the right eye.

**Figure 7:**
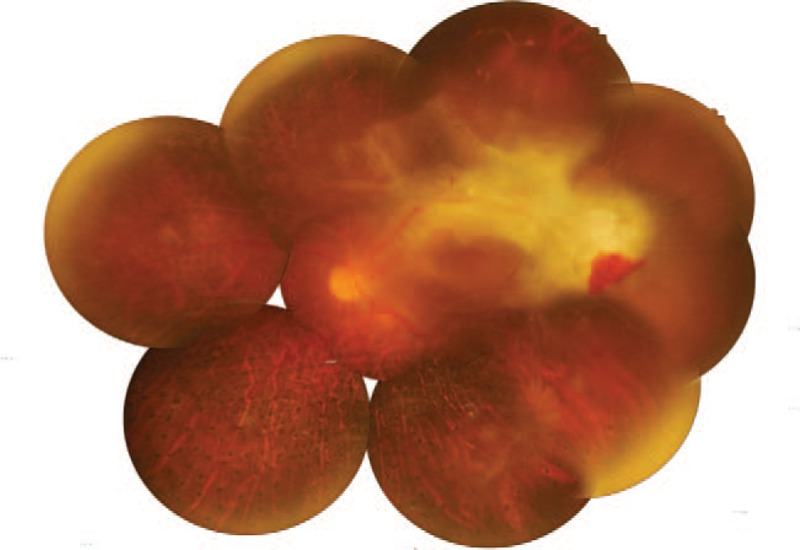
Fundus photography of the left eye revealed that the fibrovascular membrane became so extensive and enlarged to the macula.

**Figure 8:**
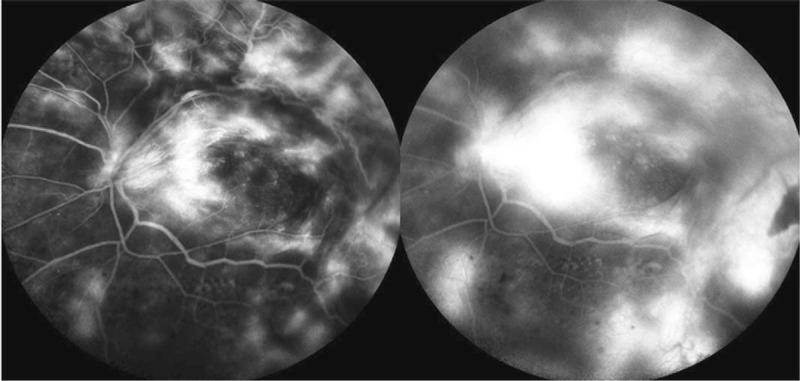
FA showed severe neovascularization of the preretinal membrane, and extensive leakage of dye from persistent new vessels.

**Figure 9:**
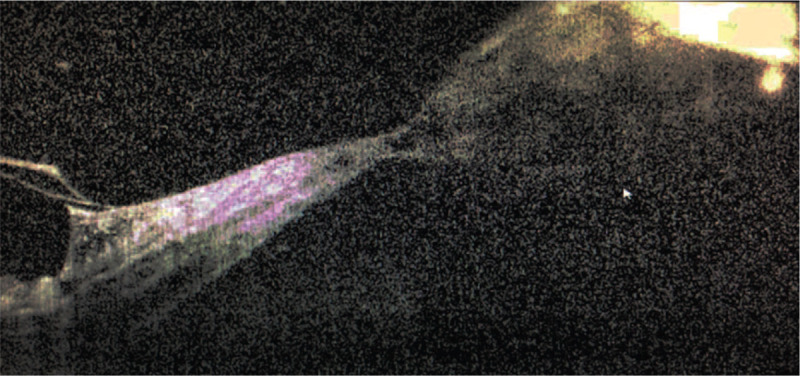
OCT revealed the TRD in the macula zone.

The patient lost to follow-up after the operation, until 2 months later, presenting with a progressive visual lost in her left eye. Upon ophthalmologic examination, the BCVA increased to 20/160 in the right eye however dropped to 20/1000 in the left. Ocular examination indicated the normal condition of bilateral anterior segment. Fundus photography and FA demonstrated the stable condition of the right eye (Fig. 6) but a rapid deterioration of the left. Fundus photography of the left eye revealed that the fibrovascular membrane became so extensive, and enlarged to the macula (Fig. 7). FA showed severe neovascularization of the fibroproliferative membrane, and extensive leakage of dye from persistent new vessels (Fig. 8). OCT depicted the TRD in the macula zone (Fig. 9). Laboratory examination showed that her HbA1c level reduced to 7.3% in just 2 months. She confessed that, in the past 2 months, she had undertaken insulin IT in local hospital with multiple insulin injections daily (Asparaginyl insulin 6 international units before meals and Glargine insulin 16 international units before sleep). And at the time of hospitalization in local hospital, she noted the decline in visual acuity in her left eye and received focal laser photocoagulation to the left eye for once. However, the visual acuity in her left eye continued to reduce. After being admitted to our hospital on 17th of April 2018 for the second time, she received intravitreal injection of conbercept (IVC) (KH902; Chengdu Kanghong Biotech Co, Ltd) to her left eye to reduce the intraoperative and postoperative incidence of hemorrhage, and 5 days later, underwent PPV in her left eye, combined with PRP and silicon oil injection. The postoperative BCVA of the left eye was 20/200 and improved to 20/160 one month later. During the subsequent 2 months of follow-up, her BCVA remained 20/160 in both eyes.

## 3 Discussion

In the long run, insulin therapy and strict blood glucose control do have positive and beneficial effects not only on the eyes, but also on other important organs of the whole body. However, studies in type 1^[2,4,5]^ and type 2^[6,7,10]^ diabetes patients have confirmed that, in comparison with conventional therapy, using insulin IT to reduce glycemic levels rapidly can bring about the onset of EWDR. And the patients at relatively more advanced stages had a greater tendency to develop EWDR than patients with mild or no DR. Although the worsening caused by IT was claimed to be temporary and recoverable, the patients being enrolled in these studies were mostly with NPDR at baseline, and PDR were all excluded. Therefore, it is not clear whether the impact of EWDR on PDR will bring serious consequences. Meanwhile the time interval of follow up being set in these trials were between 3 months to annual or even in years, and no early eye examinations were performed. The identified risk factors for EWDR were long duration of diabetes, DR severity before IT, and high initial HbA1c levels which reflects poor glycemic control.^[11]^ In addition, the magnitude of HbA1c reduction was reported to be associated with DR deterioration.^[11]^ In our case, after rapid improvement of blood glucose (with HbA1c reduction 3.7%), a severer aggravation of DR in the patient with initial high-risk PDR was observed on the second month of insulin IT. Did that mean the EWDR can be much earlier in progression and fiercer in modality in patients with pre-existing PDR? Another point to note is that, although the pathophysiology is still unclear, the insulin-like growth factor (IGF)-1 was found to play an important part in the progression of DR.^[11]^ There were hypothesis being put forward and proved in some studies that, insulin analogues, especially the ones with high affinity for IGF-1 receptor, may accelerate progression of diabetic retinopathy.^[11]^ The affinity for IGF-1 receptor was 641% for Glargine, 81% for Aspart, 20% to 25% for Glulisine and 16% for Detemi.^[12]^ The insulin analogues applied to the IT in our case were Asparaginyl and Glargine, which may also be one of the reasons leading to the acute progression of DR in the patient. Interestingly, given that sufficient laser was performed, the condition of the patient's right eye was improved in spite of the intervention of IT, which indicated the PRP may prevent the progression of DR induced by IT. Therefore, for the patients with a long diabetic history, poor glycemic control, and a pre-existing PDR above all, the control of blood glucose and the use of insulin should be more stable and prudent. If the insulin IT is inevitable, an immediate PRP should be performed before IT, and a closer eye monitoring was necessary during IT. Meanwhile the insulin analogues used in IT should be carefully selected.

## Author contributions

**Data curation:** Shuya Wang, Xiaoyan Zhang.

**Investigation:** Dezheng Liu.

**Project administration:** Hongsheng Bi.

**Supervision:** Hongsheng Bi.

**Writing – original draft:** Shuya Wang.
